# Role of New Anatomy, Biliopancreatic Reflux, and Helicobacter Pylori Status in Postgastrectomy Stump Cancer

**DOI:** 10.3390/jcm11061498

**Published:** 2022-03-09

**Authors:** Luigi Basso, Gaetano Gallo, Daniele Biacchi, Maria Vittoria Carati, Giuseppe Cavallaro, Luca Esposito, Andrea Giuliani, Luciano Izzo, Paolo Izzo, Antonietta Lamazza, Andrea Polistena, Mariarita Tarallo, Alessandro Micarelli, Enrico Fiori

**Affiliations:** 1 “Pietro Valdoni” Department of Surgery, Policlinico “Umberto I”, “Sapienza” University of Rome, 00161 Rome, Italy; daniele.biacchi@uniroma1.it (D.B.); mariavittoria.carati@uniroma1.it (M.V.C.); giuseppe.cavallaro@uniroma1.it (G.C.); lcu.esposito@gmail.com (L.E.); andrea.giuliani@uniroma1.it (A.G.); luciano.izzo@uniroma1.it (L.I.); p_izzo@hotmail.it (P.I.); antonietta.lamazza@uniroma1.it (A.L.); andrea.polistena@uniroma1.it (A.P.); mariarita.tarallo@uniroma1.it (M.T.); enrico.fiori@uniroma1.it (E.F.); 2 Department of Medicine, Surgery and Neurosciences, Operative Unit of General Surgery and Surgical Oncology, University of Siena, 53100 Siena, Italy; 3 ITER Center for Balance and Rehabilitation Research (ICBRR), 02032 Rome, Italy; alessandromicarelli@yahoo.it; 4 Eurac Research, Institute of Mountain Emergency Medicine, 39100 Bolzano, Italy

**Keywords:** gastric cancer, biliopancreatic reflux, gastritis, carcinoma, endoscopic surveillance, Helicobacter pylori infection, gastric stump cancer

## Abstract

Distal gastrectomy for benign gastroduodenal peptic disease has become rare, but it still represents a widely adopted procedure for advanced and, in some countries, even for early distal gastric cancer. Survival rates following surgery for gastric malignancy are constantly improving, hence the residual mucosa of the gastric stump is exposed for a prolonged period to biliopancreatic reflux and, possibly, to Helicobacter pylori (HP) infection. Biliopancreatic reflux and HP infection are considered responsible for gastritis and metachronous carcinoma in the gastric stump after oncologic surgery. For gastrectomy patients, in addition to eradication treatment for cases that are already HP positive, endoscopic surveillance should also be recommended, for prompt surveillance and detection in the residual mucosa of any metaplastic-atrophic-dysplastic features following surgery.

## 1. Introduction

In 1881, the Viennese surgeon Theodor Billroth and his colleagues, following previous experimental studies, successfully performed the first distal gastrectomy on a 43-year-old patient with cancer of the pylorus. Surgical reconstruction of the continuity between the residual stomach and the duodenum (gastroduodenostomy) was called “Billroth 1” (B1); three years later, Billroth experimented another technique involving anastomosis of the residual stomach to one of the first loops of the jejunum (gastrojejunostomy, Billroth 2 or B2) [1]. Both procedures were not widely adopted until the end of the 19th century: while Billroth never published his own results, these were released by one of his scholars, Wolfler, who is considered the inventor of the gastrojejunostomy antecolic technique [2]. In the following years, both techniques became universally adopted, with some geographical differences, to treat both benign and malignant gastroduodenal conditions of the distal stomach. In Asia, B1 was preferred to B2 [3], whereas in the West, and especially in Europe, B2 became more popular [4]. Since the mid-1970s, particularly in more developed countries, gastric resection has progressively become less common to cure gastroduodenal peptic ulcer disease, except for complicated cases, thanks to the diffusion of effective anti-ulcer drugs (such as H-2 receptor antagonists and, later, proton pump inhibitors). More recently, with the identification of Helicobacter pylori (HP) as a pathogen for gastroduodenal peptic disorders [5] and the introduction of specific medical treatments, surgical intervention has become even less indicated [6,7,8]. The present study is a non-systematic, narrative review of the fate of gastric resected patients, examining the role of gastric stump and of new anatomy, enteric reflux, and HP status. In non-systematic reviews there is always a risk of selection bias, including arbitrary weighing, choice and inclusion of the reported research, the way these studies are emphasized, assessed and presented, and the difficulty in integrating complex results. Nevertheless, the following is still meant to be an exhaustive and informative, albeit non-systematic, review, certainly not covering every aspect, yet still important for definition of the problem and for stressing the role of HP and of the new local environment toward the development of further malignancy, which is of potentially tremendous beneficial clinical impact.

### 1.1. Gastric Stump Cancer after Partial Gastrectomy: Role of Time

In 1922, Donald C. Balfour first noted that one of the factors which reduced life expectancy after gastric resection for peptic ulcer was later development of cancer in the residual gastric stump [9]. Since the 1950s the number of observations of “gastric stump cancer” (GSC) following B1 or B2 gastric resection increased progressively [10,11]. Thanks to the success of medical treatment of benign peptic conditions, GSC following benign situations has been decreasing, while an increase in GSC has been observed, especially in Asia, due to a larger number of gastric carcinomas operated in the early stage (early gastric cancer or EGC), translating into longer life expectancy [12,13]. In a series of 108 cases of GSC following distal gastrectomy for gastric cancer, 7 were diagnosed between 1970–1980, 28 between 1981–1991, and 73 between 1992–2002 [12]. Originally, GSC was defined as cancer arising from the remnant stomach five years after distal gastrectomy for benign disease, while later, this definition included cancers arising up to 10 years following distal gastrectomy for malignancy. This broader interval was due to the prolonged life expectancy of patients operated on for stomach cancer, and it was established that GSC is a metachronous occurrence, thus excluding the possibility of a locally recurring malignancy [14,15]. Indeed, it has been demonstrated that GSC is, on average, found 300 months after resection for benign gastroduodenal diseases and 100 months following resection for gastric cancer [12,16,17,18,19,20,21,22,23]. The type of reconstructive surgery also influences the interval of development of GSC, which occurs 84 months (mean) after B1, and 276 months (mean) after B2 (*p* < 0.01) [18]. There are, however, conflicting observations. For instance, Tokudome et al. [24] found that the ratio between observed and expected deaths from GSC was <1, and no difference was found in the cause of death in relation to the type of reconstruction performed. Asano et al. followed up for 13.1 years (mean) 6662 gastric-resected patients, finding a lower risk of death from carcinoma than in the general population, regardless of the primary disease and of the adopted surgical procedure [25]. Despite these data, distal gastrectomy is considered a precancerous condition and it represents a model to be used to study gastric carcinogenesis. In addition to time from surgery, other features have also been shown to play some role and are still investigated.

### 1.2. Gastric Stump Cancer: Role of Type of Reconstruction and of Biliopancreatic Reflux

Following distal gastrectomy, the ideal reconstruction of the intestinal continuity should not create immediate or long-term post-operative problems, nor should it alter quality of life, although alterations of the natural anatomic and functional gastric conditions cannot be avoided. B2 reconstruction is the most popular surgical procedure, but it involves a greater risk of carcinoma. Caygill et al. [26] and Toftgaard [27] in two different studies on 4466 and on 4131 patients, respectively, receiving partial gastrectomy for peptic ulcer, found that the risk of GSC was greater in those with a B2 reconstruction compared to B1 patients. In another study, the incidence of GSC in male patients younger than 40 years was four times higher after B2 than a after B1 [28]. B2 implies close exposure of the anastomosis and of the residual gastric mucosa to biliopancreatic secretions and favors gastroesophageal reflux. B1, on the other hand, preserves the physiological continuity of the duodenum, but suppression of the pyloric function may cause reflux of biliopancreatic secretions into the gastric stump, even if at a lower degree than in B2. In order to minimize reflux, B2 has been modified by connecting the afferent and the efferent loops of the jejunum (Braun’s anastomosis), a few centimeters away from the gastro-jejunal anastomosis. However, functional studies [29,30] have shown that B2 associated with Braun’s anastomosis is ineffective in preventing biliopancreatic reflux into the gastric stump, both in fasting conditions and after fatty meals. Further surgical procedures were later proposed and performed: gastro-jejunal reconstruction Roux-en-Y (R-Y), and reconstruction involving the interposition of a jejunal loop between the gastric and the duodenal stumps (jejunal interposition bilio-duodenal anastomosis, J-I). Both the R-Y and J-I procedures significantly decrease biliopancreatic reflux, which, however, to some extent still persists [31]. In any case, a 50–60 cm long R-Y loop is considered the most effective solution to reduce this problem [32]. Indeed, experimental and clinical studies have consistently demonstrated the role of reflux of duodenal contents (both bile and pancreatic juice) in the carcinogenesis of GSC [33,34,35,36,37] or, at least, as a cause of histological alterations considered to be precursors of cancer [38,39]. The carcinogenic effect of biliopancreatic reflux on the residual gastric mucosa is also indirect, due to: (1) increased alkalinity of the gastric stump caused by atrophy of the mucosa (following absence of the trophic action of antral gastrin), (2) surgical suppression of the vagal intramucosal innervation, and (3) alkaline reflux which favors growth of anaerobic nitrate-reducing bacteria. As a result, the nitrites produced by these bacteria can form cancerogenic substances when in contact with alimentary proteins [4,40]. All these changes in the microenvironment of the stump trigger chronic inflammation in the remnant mucosa, its severity gradually decreasing away from the anastomosis [41]. According to some authors, this mechanism seems to be particularly significant in carcinogenesis after B2 reconstruction, as precancerous lesions and cancer are often found at the anastomosis [19,42]. Hammar [43] analyzed the primary location of gastric carcinoma and precancerous alterations in the gastric stump of 56 B2 and 5 B1 patients. The most frequent site of GSC following B2 was right at the anastomosis. Regardless of the adopted technique of reconstruction, however, the location of GSC was often not at the anastomosis but at a site between the lesser curve and the posterior wall of the stump, corresponding to the location of the primary carcinoma of the proximal third of the stomach (PUGC) [18]. These findings suggest that pre-existing atrophic-metaplastic alterations of the mucosa, rather than biliopancreatic reflux, are the most likely cause of GSC [12]. As suggested by Kondo [44], GSC after gastrectomy for cancer, in addition to arising earlier after surgery, is non-anastomotic when compared to GSC after resection for benign diseases.

### 1.3. Gastric Stump Cancer: The Issue of Synchronous Multiple Gastric Cancers

Patients receiving surgical treatment for cancer already harbor, at the time of their surgery, alterations in the residual gastric mucosa which can have some relationship with primary cancer. The importance of synchronous multiple gastric cancers has also been highlighted. Fujita et al. [13] noted a higher incidence of GSC in patients with less differentiated synchronous multiple gastric cancers at primary surgery, compared to patients with single well-differentiated tumors (respectively *p* < 0.05 and *p* < 0.05). Nevertheless, in two series of 639 patients with distal EGC, secondary lesions were rarely found and no patient with distal EGC showed a secondary synchronous cancer in the upper third of the stomach [45,46]. Once again, although there are similar features in PUGC and GSC, both arising in the same gastric mucosa area, the incidence of PUGC decreases parallel to the incidence of gastric carcinoma in general, although PUGC represents only 3–4% of all gastric malignancies [47,48,49,50]. Some authors have shown that a few genetic alterations cause gastric metaplastic lesions, which are considered precursors of cancer, while others influence the proliferation and aggressiveness of gastric cancer [51,52,53,54,55]. It has been hypothesized that the onset of GSC can be related to these genetic predispositions [13]. Matsui et al. suggested that reflux is the major cause of GSC in patients receiving hemigastrectomy for benign disease, whereas, in those operated for cancer, if GSC appears ≥10 years later, this could be related to genetic factors (such as p53), responsible for multiple metachronous carcinogenesis, while if GSC occurs <10 years, this new cancer could be caused by diffuse metaplastic lesions of the residual mucosa [56]. Molecular studies [57,58] have shown an incidence of micro satellite instability in patients with GSC higher than in those with PUGC. It is impossible to verify the existence of residual malignant cells or potentially malignant mucosal fields at the time of gastric resection. It can be hypothesized that these pathways of carcinogenesis could have remained silent and inactive if the anatomic and functional alterations produced by surgery had not occurred.

### 1.4. Gastric Stump Cancer: Role of HP Infection

The residual post-gastrectomy mucosa is considered hostile to HP; hence HP infection progressively decreases following surgery. This is due to at least three reasons: (1) the antrum, which is the HP natural environment, has been removed; (2) the increased pH due to biliopancreatic reflux inhibits HP proliferation [59,60,61]; (3) the residual mucosa is replaced by an infection-resistant atrophic-metaplastic epithelium [62]. Some authors think that the spontaneous decrease in the infection starts at the anastomosis, where the gastric mucosa changes from the characteristics of infective chronic active gastritis to those of reflux gastritis (foveolar hyperplasia, congestion, paucity of inflammatory infiltrate, glandular cystic dilatation) [43]. Suh et al. have shown the spontaneous disappearance of infection in 38.6% of 70 patients within 18 months from distal gastrectomy [63], while in another study [60], the prevalence of HP infection varied over time following surgery, being 29.5% less than 25 years after gastric resection, 13.6% from 16 to 30 years after surgery, and 10% > 30 years later. In the published literature, however, the rates of gastric stump infection fall within a broad range. Indeed, according to some authors, overall HP infection occurs in 50–68.2% of distal-gastrectomy patients, in 55–72% of B1 patients, in 58–66% of B2, and in 26% of patients reconstructed by R-Y surgery [31,60,62,64]. In a review of 36 studies on partial gastrectomy for gastric ulcer, HP infection occurred in 50% of cases after surgery (range from 19% to 73%) [65]. In another research, HP infection rate was 71% after B1, and 46% after B2 [66]. Other authors have confirmed HP infection rates in patients treated by B2 lower than those observed in subjects treated by B1 or R-Y reconstruction [66,67]. Chan et al. [31] showed that R-Y reconstruction causes less reflux during fasting and in the postprandial period, and a lower incidence of HP gastric stump infection than B2 reconstruction, even when B2 is associated with Braun’s anastomosis. These conflicting results can be explained by differences in HP infection diagnostic methods in resected stomachs. In these instances, diagnosis of HP infection is less accurate with the urease breath test (UBT) than histology, while the rapid urease test (RUT) is more accurate than histology [68]. However, it should also be considered that the accuracy of biopsies is altered by patchy and uneven distribution of HP infection in the gastric mucosa [69] and by the number of biopsies taken. As reported by Chun et al. [70], following partial gastrectomy for cancer, UBT was comparable to RUT in terms of accuracy (UBT 87%, RUT 72%). High levels of anti-HP antibodies can be found in the serum, even when infection is not detected by microscopic examination or by culture methods [64]. Diagnosis of infection using enzyme immunoassay for HP antigen in stools appears to be a highly reliable test in gastrectomy patients, capable of detecting both the presence of infection and the success of post-treatment HP eradication [71]. Furthermore, based on the accepted role of HP infection in gastric carcinogenesis [72,73,74] (HP infection being found in 54–71% of cases of primitive gastric carcinoma), it has been hypothesized that HP eradication in gastrectomy patients could prevent the development of GSC. The Maastricht IV/ Florence Consensus Report and the Second Asia-Pacific Consensus Guidelines both recommend eradication of HP infection from the gastric stump [75,76]. However, it has also been highlighted that the role of the microorganism in the development of GSC is different from primitive gastric cancer in the intact stomach, where HP promotes carcinogenesis through the cytotoxin-associated gene A or CagA protein, which acts as a growth factor for the cells of the gastric mucosa [77,78]. Due to the reduced levels of the microorganism in the partial-gastrectomy patients, it is unlikely that HP plays here the same carcinogenic role as in the intact stomach [59,79,80,81,82]. There are data, however, confirming the importance of HP infection also in the development of gastritis in the gastric stump. Our research group has shown that in 151 partial-gastrectomy peptic ulcer patients, after a mean interval of 25 years from surgery, there was a 10-fold increase in the prevalence of normal mucosa in HP-negative (22.0%) vs. HP-positive (2.4%) patients, and the prevalence of intestinal metaplasia was four times higher in HP-positive than in HP-negative patients (19.6% vs 4.6%) [83]. In another endoscopic study assessing 187 peptic ulcer hemi-gastrectomy patients (mean interval from surgery = 27.8 years) or distal gastric cancer patients (mean interval from surgery = 7.6 years), we observed that chronic atrophic gastritis, intestinal metaplasia, and dysplasia are more common in the HP-positive group (OR 2.37, *p* = 0.007) [84]. However, HP-positive patients resected for cancer showed a higher risk of atrophic/metaplastic/dysplastic lesions compared to both HP-negative cancer patients (OR 4.20) and to HP-negative and HP-positive patients resected for peptic ulcer (OR 1.59). The concentration of interleukin (IL)-8, a marker for inflammation, in the residual gastric mucosa three months after surgery, was significantly higher in B1-B2 than in R-Y reconstruction and in HP-positive compared to HP-negative patients.

### 1.5. Gastric Stump Cancer: Combined and Synergic Role of HP Infection and of Biliopancreatic Reflux

Biliopancreatic reflux and HP infection are considered independent risk factors for the development of gastritis-metaplastic lesions of the gastric stump [85]. Hamaguchi et al. [86] studied 12 cases of gastrectomy with B1 reconstruction for EGC resulting HP-positive after resection; one- and six-months after eradication, significant improvements of the mucosal erythema at endoscopy (*p* = 0.038) and of the gastritis activity at histology (*p* < 0.0001) were observed compared to pre-eradication findings. In another study on eight patients with B1 reconstruction for carcinoma, following confirmed UBT eradication, the microscopic features of chronic inflammation improved progressively over a period of 9 years in both greater and lesser curves of the gastric stump. The improvement of these potentially precancerous alterations might inhibit the development of metachronous cancer [87]. However, in resected patients, there is a possible synergic role of both biliopancreatic reflux and HP infection in the development of lesions of gastric mucosa. The relative importance of each of them in the development of metaplastic-atrophic lesions has not yet been established, and it is likely that these mucosal alterations, representing a morphological point of no return, may not be repaired by eradication alone [88]. In HP-positive patients, infection is one of the risk factors contributing to carcinogenesis, and its eradication decreases the damage to the gastric mucosa, mainly by reducing inflammation. There is no guarantee of a similar positive effects for metaplastic-atrophic lesions [89,90,91,92], and simple improvement of some features of mucosal inflammation might not suffice to prevent metachronous carcinoma [93]. Johannesson et al. [81] studied 29 partial gastrectomy patients (5 B1 and 24 B2). These patients were re-resected with a R-Y reconstruction, due to reflux gastritis or to severe dysplasia/EGC, at a median interval of 19 years from index surgery. The aim of this research was to investigate the effect of bile diversion and of HP infection on histological features of anastomotic biopsies taken 5–17 years after re-operation. Regarding the surgical specimen of re-resection, the follow-up biopsies showed an unchanged prevalence of infection but an increase in active chronic gastritis, atrophy, intestinal metaplasia, and dysplasia. The HP status, however, had no effect on the progression of active chronic gastritis, atrophy, and intestinal metaplasia. A significant increase in dysplasia as such was observed in HP-positive patients, while prevalence of both moderate and severe grades of dysplasia were not related to HP status. In any case, a significant reduction of gastric carcinoma was achieved in non-operated stomachs by the eradication of HP infection, as long as metaplastic-atrophic-dysplastic lesions were absent [88]. Therefore, eradication should be completed rapidly to inhibit the development and progression of pre-cancerous lesions [94,95]. GSC may occur even in patients with resected stomachs as part of pancreatoduodenectomy for malignant disease. Pflüger et al. found six cases of GSC out of 4414 cases treated at their institution from 2000 until 2015 for pancreatic malignancy [96]. Nevertheless, GSC in these situations is rare, simply because of the grim prognosis and short life expectancy of these patients. 

### 1.6. HP Infection and Metachronous Carcinoma following Endoscopic Resection of EGC

There are no data confirming the beneficial role of HP eradication when comparing metachronous gastric cancer in eradicated vs. non-eradicated HP-positive partial-gastrectomy cancer patients, while there are interesting data on the impact of eradication of HP infection on the incidence of metachronous gastric cancer following endoscopic resection of EGC. After endoscopic resection, the residual gastric mucosa represents a potential site for metaplastic-atrophic lesions. In these circumstances, metachronous carcinomas may occur more frequently, while it cannot be ruled out that undetected synchronous cancers, already present, are left untreated. In a non-randomized study on 132 HP-seropositive patients after endoscopic resection of EGC, Uemura et al. [97] completed oral HP eradication therapy on 65 of these 132. In the treated group, endoscopic biopsies from the antrum and the greater curvature 6 months after eradication showed a significant reduction of neutrophilic infiltration (*p* < 0.01) and of the severity of metaplasia (*p* < 0.05), while in the non-treated group (67 patients), there were six cases of metachronous gastric cancer over a period of 48 months, compared to no cases in the treated group (*p* < 0.01). In a retrospective multicenter study, Nakagawa et al. [98] analyzed the incidence of metachronous gastric carcinoma in 2835 patients endoscopically resected for EGC. At a mean follow-up of 2 years, metachronous carcinoma was found in 5% of 2469 non-HP eradicated patients and in 2% of 356 successfully eradicated subjects (*p* = 0.021). In a series of 176 patients with EGC endoscopically treated [99], nine cases of metachronous gastric carcinoma were detected after a mean period of 30 months following treatment. In the univariate analysis, age >70 years (*p* = 0.015) and the presence of severe mucosal atrophy of the gastric body (*p* = 0.031) and antrum (*p* = 0.008) were significantly linked to metachronous malignancy. In the multivariate analysis, the degree of antrum atrophy was confirmed as an independent risk factor for metachronous carcinoma (*p* = 0.011). In relation to HP infection, four metachronous gastric cancers were found in 94 patients to have been successfully eradicated after endoscopic resection, and in 2 of 22 non-treated patients (*p* = not significant). In a multicenter, open label, randomized controlled trial [100], Fukase et al. analyzed the prophylactic role of HP eradication on the incidence of metachronous gastric carcinoma following endoscopic resection for EGC. In the studied population of 505 patients, 255 belonged to the eradication group (203 of whom had their cancer successfully eradicated), and 250 patients were controls. During a 3-year follow-up after endoscopic resection, metachronous carcinoma developed in 9 of the 203 eradicated patients and in 24 of the 250 controls (HR 0.399 *p* = 0.003). Similar trends were reported by Maehata et al. [93] in 268 patients followed-up for a mean period of 3 years after endoscopic resection for EGC. Over an 11-year term, metachronous gastric carcinomas were detected in 13 of 91 non-successfully eradicated patients, and in 15 of 177 successfully eradicated subjects (*p* = not significant). In 10 of the 15 eradicated patients who developed metachronous gastric cancer, this developed more than 5 years after endoscopic resection of the primary malignancy. In the multivariate analysis, in addition to the post-treatment interval, an independent risk factor for metachronous gastric carcinoma was represented by severe gastric atrophy at the time of endoscopic resection. These results suggest that HP eradication delays but does not completely protect against later development of a malignancy if the residual mucosa already suffers from atrophic lesions. Besides, another study on patients endoscopically resected for EGC, found no significant differences in metachronous carcinoma between 263 eradicated and 105 non-eradicated patients, over a period of 60 months [101]. More recently, Li and Yu, reviewing 15 years of literature, suggested that eradication of HP infection after endoscopic resection of EGC could reduce the incidence of metachronous precancerous lesions and of metachronous gastric cancer [102]. Hence, the role of HP in chronic gastritis, peptic ulcer disease and gastric cancer is, once again, confirmed, while its importance for many types of extra-gastric disease still remains poorly researched [103]. 

## 2. Conclusions

Following distal gastric resection, the microenvironment of the gastric stump changes dramatically, due to both the new anatomy and to the consequences of surgery on the residual gastric stump, which favor biliopancreatic reflux. The presence of HP infection gradually decreases following surgery but contributes, together with reflux, to damage the residual gastric mucosa. Under these influences, the gastric epithelium becomes a site for metaplastic-atrophic lesions, referable to either the pre- or the post-operative phases, which can be considered as precursors for cancer. Eradication of HP infection can prevent or delay both the development of precancerous lesions and their favoring role on carcinogenesis. There is a lack of studies evaluating the effect of eradication therapy in preventing GSC in hemi-gastrectomy cancer patients, HP-positives after surgery. As we have already emphasized, ours is only a non-systematic, narrative review of the local outcome of gastric resected patients, and, therefore, our research is probably biased, at least to some extent. Nevertheless, we would like to stress that our purpose was to provide an exhaustive and informative review, still important for definition of the problem and for stressing the role of HP and of the new local environment in the development of further malignancy, which has important clinical implications.
